# Effects of Iloprost on Arterial Oxygenation and Lung Mechanics during One-Lung Ventilation in Supine-Positioned Patients: A Randomized Controlled Study

**DOI:** 10.3390/jpm12071054

**Published:** 2022-06-27

**Authors:** Kyuho Lee, Mina Kim, Namo Kim, Su Jeong Kang, Young Jun Oh

**Affiliations:** 1 Department of Anesthesiology and Pain Medicine, Yonsei University College of Medicine, Seoul 03722, Korea; theoneimlee@yuhs.ac (K.L.); namo@yuhs.ac (N.K.); kangsuda@yuhs.ac (S.J.K.); 2 Anesthesia and Pain Research Institute, Yonsei University College of Medicine, Seoul 03722, Korea; 3 Department of Anesthesiology and Pain Medicine, Dongguk University Ilsan Hospital, Goyang-si 10326, Gyeonggi-do, Korea; exrexr20@gmail.com

**Keywords:** one-lung ventilation, supine position, iloprost, oxygenation, lung mechanics

## Abstract

Patients undergoing one-lung ventilation (OLV) in the supine position face an increased risk of intraoperative hypoxia compared with those in the lateral decubitus position. We hypothesized that iloprost (ILO) inhalation improves arterial oxygenation and lung mechanics. Sixty-four patients were enrolled and allocated to either the ILO or control group (*n* = 32 each), to whom ILO or normal saline was administered. The partial pressure of the arterial oxygen/fraction of inspired oxygen (PaO_2_/FiO_2_) ratio, dynamic compliance, alveolar dead space, and hemodynamic variables were assessed 20 min after anesthesia induction with both lungs ventilated (T1) and 20 min after drug nebulization in OLV (T2). A linear mixed model adjusted for group and time was used to analyze repeated variables. While the alveolar dead space remained unchanged in the ILO group, it increased at T2 in the control group (*n* = 30 each) (*p* = 0.002). No significant differences were observed in the heart rate, mean blood pressure, PaO_2_/FiO_2_ ratio, or dynamic compliance in either group. Selective ILO nebulization was inadequate to enhance oxygenation parameters during OLV in the supine position. However, it favorably affected alveolar ventilation during OLV in supine-positioned patients without adverse hemodynamic effects.

## 1. Introduction

One-lung ventilation (OLV) is an essential part of thoracic anesthesia which allows access to the surgical field [1]. However, inevitable development of intrapulmonary shunt during OLV makes the maintenance of adequate oxygenation a major issue for this ventilation technique [2]. Arterial oxygenation during OLV is affected by the distribution of pulmonary perfusion to the ventilated and non-ventilated lungs [3]. Hypoxemia induced by collapse of one lung activates hypoxic pulmonary vasoconstriction, which reduces intrapulmonary shunt by redirecting pulmonary perfusion to the well-ventilated lung [4].

Along with hypoxic pulmonary vasoconstriction, body position may further affect the distribution of pulmonary perfusion [3]. Most thoracic surgeries are performed in the lateral decubitus position, and gravity induces better perfusion of the lower, ventilated lung than that of the upper, non-ventilated lung [3]. However, surgeries involving the anterior mediastinum often require OLV in the supine position, in which favorable gravitational modulation of pulmonary perfusion cannot be anticipated [5]. Hence, patients scheduled for these surgeries face an increased risk of hypoxemia during OLV [6].

Iloprost (ILO) is a prostacyclin analogue, and inhalation of the drug induces vasodilation in the well-ventilated areas of the lung with little effect on systemic circulation [7]. Hence, recent studies have investigated the capability of ILO as a potential rescue drug for hypoxia during OLV, and demonstrated that selective ILO nebulization to the ventilated lung improved arterial oxygenation and lung mechanics in pulmonary resections [8,9,10]. However, whether ILO would provide similar effects during OLV in the supine position is not known. Hence, we hypothesized that ILO would improve arterial oxygenation and lung mechanics in patients scheduled for anterior mediastinal mass excision and investigated the effects of ILO in this cohort. 

## 2. Materials and Methods

### 2.1. Study Population 

This prospective, randomized, controlled study included patients scheduled for video-assisted thoracoscopic anterior mediastinal mass excision between July 2021 and March 2022 and adhered to the Consolidated Standards of Reporting Trials (CONSORT) guidelines. The study was approved by the Institutional Review Board of Severance Hospital, Yonsei University Health System, Seoul, Korea (IRB, no. 4-2021-0694) and was registered at Clinicaltrials.gov (NCT 04927039). After IRB approval, written informed consent was obtained from all participants involved in the study, and the study methods were performed in accordance with the relevant guidelines and regulations. The inclusion criteria were as follows: (1) scheduled for video-assisted thoracoscopic mediastinal mass excision requiring OLV, (2) aged between 20 and 80 years, and (3) American Society of Anesthesiologists physical status class between II and III. The exclusion criteria included morbid obesity, heart failure (New York Heart Association class III or IV), arrhythmia, and severe hepatic, renal or pulmonary diseases. 

### 2.2. Anesthetic Management

Anesthesia was induced using propofol (1.0–2.0 mg/kg), remifentanil (0.5–1.0 μg/kg), and rocuronium (0.8–1.0 mg/kg). Patients were intubated with left-sided double-lumen endobronchial tubes (DLT) (VentiBronc^TM^ Anchor; Flexicare Medical Ltd., Mountain Ash, UK). A fiberoptic bronchoscope was used to confirm the correct position of the DLT before the OLV was provided. The radial artery was cannulated for continuous pressure monitoring and arterial blood gas analysis. Mechanical ventilation was provided using autoflow pressure-controlled ventilation mode (Primus^®^; Dräger Medical, Lubeck, Germany). The fraction of inspired oxygen (FiO_2_) was set at 0.6. The tidal volume was adjusted to 6 mL/kg, and a positive end-expiratory pressure (PEEP) of 5 mm Hg was applied. The respiratory rate was adjusted to maintain the end-tidal carbon dioxide (etCO_2_) at the range of 35–40 mmHg. Anesthesia was maintained with 1.0–2.0 vol% sevoflurane and 0.1–0.3 μg/kg/min remifentanil, and the depth of anesthesia was assessed using a Sedline^®^ brain function monitor (Masimo, Irvine, CA, USA). The rate of intravenous fluid administration was set at a rate of 3 mL/kg/h, and additional fluid was administered to compensate for intraoperative blood loss. 

After anesthesia induction, OLV was initiated. Carbon dioxide (CO_2_) insufflation was initiated on the operating side of the thorax by the surgeon with a pressure limit of 10 mmHg and a flow rate of 4 L/min. Vasoactive drugs, such as phenylephrine, were administered if systolic blood pressure (SBP) fell below 80 mmHg. In cases of desaturation (saturation of percutaneous oxygen [SpO_2_] < 95%), the FiO_2_ level increased by 0.2 up to 1.0. 

### 2.3. Study Design and Outcome Measurements

All enrolled patients were randomly allocated to either the ILO or the control group using a randomized sequence, and surgeons and anesthesiologists were blinded to the group allocation. After the initiation of OLV, ILO (20 μg (2 mL, Ventavis^®^; Bayer AG, Leverkusen, Germany) was administered to the ILO group. ILO was mixed with normal saline (3 mL) and aerosolized using an ultrasonic nebulizer (Aerogen^®^ Pro; Aerogen Ltd., Galway, Ireland), which was connected to the inspiratory limb of the ventilator system. A comparable volume (5 mL) of normal saline was nebulized to the control group in the same manner. The medications were nebulized for 20 min.

The study time points were as follows: (1) 20 min after anaesthesiainduction with both lungs ventilated (T1) and (2) 20 min after ILO or normal saline nebulization in OLV (T2). Respiratory and hemodynamic parameters were recorded and arterial blood samples were collected during each study period. Respiratory parameters included FiO_2_, etCO_2_, partial pressure of arterial oxygen (PaO_2_), the ratio of PaO_2_ to FiO_2_ (PaO_2_/FiO_2_), alveolar dead space, and dynamic compliance. A blood gas analyzer (GEM^®^ Premier 4000; Instrumentation Laboratory, Lexington, MA, USA) was used to obtain PaO_2_ and the partial pressure of arterial carbon dioxide (PaCO_2_). Dead space ventilation was calculated according to the Hardman and Aitkenhead equation as follows: (1.135 × (PaCO_2_ − etCO_2_)/PaCO_2_ − 0.005) [11]. Dynamic compliance was calculated using the following equation: (tidal volume/(plateau airway pressure-PEEP)). Hemodynamic parameters included the heart rate and arterial blood pressure. The incidence of intraoperative hypotension (SBP < 80 mmHg) and hypoxia (SpO_2_ < 90%) after the initiation of drug administration was recorded.

### 2.4. Statistical Analysis

The primary outcome was the change in PaO_2_/FiO_2_ at 20 min at T2, and the secondary outcome was the change in other respiratory mechanics such as alveolar dead space and dynamic compliance. Considering the results of a previous study in which the difference in PaO_2_/FiO_2_ ratio was 30 mmHg with a standard deviation of 30 mmHg between patients who inhaled ILO and those who did not [9], a sample size of 27 participants per group was needed to achieve a power of 0.95 and an alpha level of 0.05. Assuming a 20% dropout rate, 32 patients were enrolled in each group. 

The student’s *t*-test was used to analyze continuous variables, and the Wilcoxon signed-rank test was used to analyze variables which did not meet normality. To compare categorical variables between the groups, chi-square test or Fisher’s exact test was used. Repeated variables were analyzed using a linear mixed model with group and time and the interaction between groups and time as a fixed effect. Post hoc analysis with Bonferroni correction for within-group comparisons versus T1 and between-group comparisons versus T2 was performed for multiple comparisons. The results are expressed as mean ± standard deviation, median (interquartile range), or number (percentage). SPSS 25.0 software (IBM Corp., Armonk, NY, USA) was used for the statistical analyses, and *p* < 0.05 was considered statistically significant.

## 3. Results

**Figure 1:**
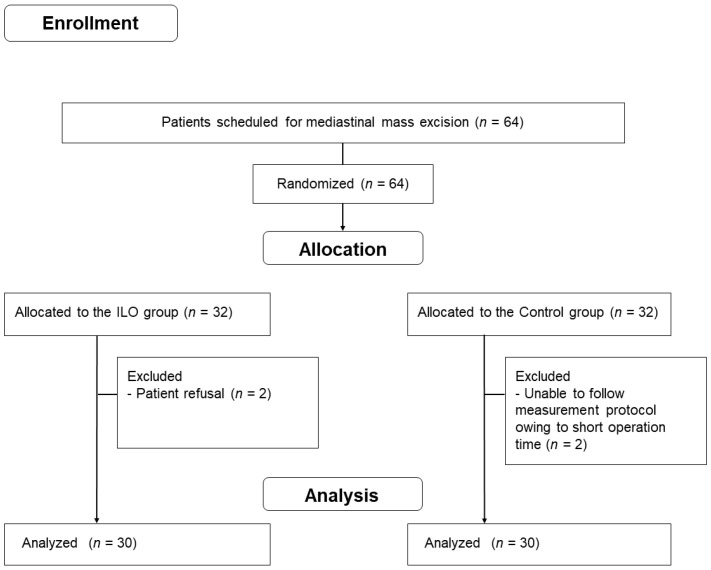
Patient enrollment.

Sixty-four patients scheduled for video-assisted thoracoscopic mediastinal mass excision were enrolled in this study (Figure 1). Two patients in the ILO group were excluded because of patient refusal, and two in the control group were excluded as the measurement protocol could not be properly executed owing to the short operation time. Hence, the data of 30 patients in each group were analyzed. 

Intergroup comparisons of the preoperative variables between the two groups are shown in Table 1. Age, sex, height, weight, body mass index, and ASA classification were similar between groups. The incidence of hypertension and diabetes mellitus, history of cigarette smoking, incidence of pulmonary abnormalities according to preoperative computed tomography, and variables derived from preoperative spirometry were also comparable between the groups, except for FEV_1_. 

Intraoperative data are presented in Table 2. All variables, including the side of the operation, anesthesia time, operation time, OLV time, incidence of intraoperative hypotension, hypoxia, fluid intake, urine output, and estimated blood loss during surgery, were comparable between the two groups, with the exception of the incidence of FiO_2_ elevation, which was more frequent in the control group (*p* = 0.006). 

Data regarding hemodynamics and lung mechanics are presented in Table 3. No clinically relevant differences were observed between the two groups at T1. At T2, PaO_2_, PaO_2_/FiO_2_ ratio, and dynamic compliance of the two groups were significantly decreased compared to those at T1; however, no statistical significance was observed in the linear mixed-model analysis adjusted for group and time. Changes in heart rate, mean blood pressure, and etCO_2_ were insignificant in both groups. While PaCO_2_ and alveolar dead space remained unchanged in the ILO group, those of the control group were significantly increased at T2 (*p* = 0.04 and 0.002, respectively). 

## 4. Discussion

In this study, we demonstrated that selective nebulization of ILO favorably affected alveolar ventilation in patients who underwent OLV in the supine position, with a significant difference in alveolar dead space observed between the ILO and control groups. However, ILO nebulization did not lead to a significant improvement in arterial oxygenation.

Inhaled ILO selectively dilates pulmonary terminal capillaries surrounded by alveoli, resulting in increased pulmonary blood flow in the well-ventilated areas of the lung [12]. Hence, the effects of ILO were recently investigated by anesthesiologists as a potential rescue drug for hypoxia during OLV in thoracic surgery [8,9,10]. Indeed, previous studies indicated that the selective ILO administration to the ventilated lung during OLV induced a decrease in ventilation/perfusion (V/Q) mismatch and improved oxygenation [8,9,10]. Yet these results were confined to patients who underwent pulmonary resection, which is performed in the lateral decubitus position. As the lateral position itself improves the V/Q match in OLV owing to gravitational forces reducing the shunt of the upper non-ventilated lung [3], it is reasonable to assume that patient positioning significantly contributed to enhanced lung mechanics and arterial oxygenation in these studies. However, it is not yet clear whether ILO would still demonstrate similar favorable effects in the OLV of supine-positioned patients, in whom favorable modulation of perfusion by gravity cannot be anticipated [5]. 

Our results indicate that ILO positively affected alveolar ventilation in the supine position, which is consistent with the results of previous studies [9,10]. While the etCO_2_ of the two groups remained unchanged since we adjusted the respiratory rate to maintain the parameter at its target range throughout the measurement periods, significant increases were observed in PaCO_2_ of the control group, leading to an increase in the alveolar dead space. In contrast, no significant change in the alveolar dead space was observed within the ILO group, indicating ameliorated alveolar ventilation induced by ILO.

Nonetheless, we did not observe a significant improvement in PaO_2_ or the PaO_2_/FiO_2_ ratio in the ILO group. Indeed, some studies reported that ILO inhalation produced similar results in patients with pulmonary hypertension secondary to chronic obstructive pulmonary disease [13,14]. However, we speculate that in those studies, non-selective administration of ILO to patients with severe cardiopulmonary diseases may have induced vasodilation not only in well-ventilated areas but also in poorly ventilated areas of the lung, which would have aggravated the V/Q mismatch. However, most of our patients were classified as ASA II without severe cardiopulmonary diseases, and since we selectively administered ILO to the ventilated lung with the aid of a double-lumen endobronchial tube, unintended vasodilation of poorly ventilated areas of the lung seems unlikely. Rather, with gravity affecting both lungs equally in the supine position, we presume that the pharmacological vasodilation confined to the ventilated lung was inadequate to decrease the shunt induced in the non-ventilated lung. Nonetheless, it is notable that the number of patients who required FiO_2_ elevation, which was triggered when SpO_2_ fell below 95%, significantly differed between the two groups (one in the ILO group and nine in the control group), implying that ILO may have contributed to oxygenation. Given that the majority of our patients exhibited normal pulmonary function and successfully endured OLV without experiencing severe hypoxia, studies involving morbid patients with pulmonary diseases undergoing OLV in the supine position are warranted to further elucidate the impact of ILO on arterial oxygenation.

Evidence indicates that ILO administration may be associated with systemic vasodilation and inhibition of platelet aggregation [15]. Indeed, we frequently observed hypotension during OLV in both groups. However, the incidence was comparable between the groups, indicating that short-term ILO nebulization was not significantly associated with intraoperative hypotension. Considering that CO_2_ was routinely insufflated in all cases to achieve a clear surgical view, we presume that induction of capnothorax may have contributed to the comparably high incidence of hypotension [16,17]. With regard to platelet function, the estimated blood loss was minor, and no significant difference was observed between the two groups, implying platelet inhibition induced by ILO to be negligible. Comprehensively, a short-term ILO nebulization seems less likely to be associated with intraoperative adverse events.

The limitations of the study are as follows: owing to the short operative time, the baseline measurement (T1) was inevitably performed during two-lung ventilation. The effects of ILO on arterial oxygenation may have been more clearly elucidated if all parameters could be measured during the OLV. However, pursuing such a measurement protocol would otherwise have compelled undesirable elongation of anesthesia time since mediastinal mass excision in our institution generally requires less than 50 min of OLV. In addition, we could not measure pulmonary shunts because pulmonary artery catheterization is not routinely performed during mediastinal mass excision. 

## 5. Conclusions

In conclusion, selective ILO nebulization was inadequate to enhance the PaO_2_/FiO_2_ ratio in healthy, supine-positioned patients. Nonetheless, it positively affected alveolar ventilation without adverse hemodynamic effects and decreased the requirement for FiO_2_ elevation during OLV. Whether ILO ameliorates arterial oxygenation in morbid patients remains to be proven in future studies.

## Figures and Tables

**Table 1:** Preoperative data.

	Control Group (*n* = 30)	ILO Group (*n* = 30)	*p*-Value
Age (yrs)	50 ± 12	53 ± 14	0.374
Women (*n*)	15 (50)	16 (53)	0.796
Height (cm)	164.4 ± 9.2	164.2 ± 11.1	0.923
Weight (kg)	67.9 ± 12.3	66.6 ± 13.9	0.705
Body mass index (kg/m^2^)	24.9 ± 3.0	24.5 ± 3.4	0.622
ASA classification 2/3 (*n*)	28 (93)/2 (7)	26 (87)/4 (13)	0.389
Hypertension (*n*)	10 (33)	12 (40)	0.592
Diabetes mellitus (*n*)	2 (7)	5 (17)	0.228
Smoking history			
Ex-smoker or current smoker (*n*)	9 (30)	14 (47)	0.184
Smoking index (pack × years)	0 [0–11]	0 [0–10]	0.412
Preoperative chest CT			
Atelectasis (*n*)	1 (3)	4 (13)	0.161
Bronchiectasis (*n*)	1 (3)	3 (10)	0.301
Emphysema (*n*)	0 (0)	2 (7)	0.150
Bronchitis (*n*)	3 (10)	1 (3)	0.301
Preoperative spirometry			
FEV_1_ (L)	2.9 ± 0.8	2.6 ± 1.1 *	0.037
FEV_1_ (% predicted)	92 [85–96]	83 [75–94]	0.368
FVC (L)	3.6 ± 1.0	3.4 ± 1.2	0.569
FVC (% predicted)	89 [83–98]	88 [77–97]	0.276
FEV_1_/FVC (%)	81 [76–84]	77 [72–81]	0.069

Data presented as mean ± standard deviation, number (%), or median [interquartile range]. ASA, American Society of Anesthesiologists; CT, computed tomography; FEV_1_, forced expiratory volume in 1 s; FVC, forced vital capacity. * *p* <0.05 vs. control group.

**Table 2:** Intraoperative data.

	Control Group (*n* = 30)	ILO Group (*n* = 30)	*p*-Value
Approach direction (right/left) (*n*)	16 (53)/14 (47)	15 (50)/15 (50)	0.796
Anesthesia time (min)	109 ± 32	116 ± 38	0.480
Operation time (min)	70 ± 29	76 ± 35	0.481
OLV time (min)	51 [42–66]	49 [40–61]	0.812
FiO_2_ elevation (*n*)	9 (30)	1 (3) *	0.006
Hypoxia (*n*)	1 (3)	1 (3)	1.000
Hypotension (*n*)	14 (47)	13 (43)	0.795
Intake fluid (mL)	702 ± 259	700 ± 302	0.982
Urine output (mL)	25 ± 45	43 ± 71	0.254
Estimated blood loss (mL)	35 ± 20	34 ± 18	0.738

Data are presented as the mean ± standard deviation, number (%), or median (interquartile range). ILO, iloprost; OLV, one-lung ventilation; FiO_2_, fraction of inspired oxygen; SpO_2_, oxygen saturation (pulse oximetry); hypotensive event defined as the incidence of systolic blood pressure < 80 mmHg; hypoxic event defined as the incidence of SpO_2_ < 90% requiring anesthetic intervention. * *p* < 0.05 vs. control group.

**Table 3:** Effects of iloprost on hemodynamics and lung mechanics.

	Control Group (*n* = 30)	ILO Group (*n* = 30)	*p*-Value
Heart rate (beat/min)			0.83
T1	72 ± 10	78 ± 14	
T2	74 ± 14	81 ± 12	
Mean blood pressure (mmHg)			0.95
T1	77 ± 11	80 ± 11	
T2	81 ± 12	85 ± 13	
PaO_2_ (mmHg)			0.77
T1	253 ± 66	255 ± 68	
T2	108 ± 37 *	115 ± 45 *	
PaO_2_/FiO_2_ ratio (mmHg)			0.29
T1	422 ± 111	425 ± 113	
T2	156 ± 42 *	190 ± 75 *	
EtCO_2_ (mmHg)			0.49
T1	39 ± 5	40 ± 4	
T2	40 ± 3	42 ± 5	
PaCO_2_ (mmHg)			0.04
T1	43 ± 5	45 ± 5	
T2	48 ± 5 *	47 ± 6	
Alveolar dead space			0.002
T1	12 ± 12	12 ± 9	
T2	19 ± 6 *	11 ± 5	
Dynamic compliance (mL/cmH_2_O)			0.41
T1	28 ± 5	27 ± 6	
T2	16 ± 3 *	16 ± 5 *	

Data are presented as the mean ± standard deviation. ILO, iloprost; T1, 20 min after initiation of two-lung ventilation; T2, 20 min after iloprost or saline administration; PaO_2_, partial pressure of arterial oxygen; PaO_2_/FiO_2_ ratio, ratio of PaO_2_ to fraction of inspired oxygen; EtCO_2_, end-tidal carbon dioxide; PaCO_2_, partial pressure of arterial carbon dioxide. *p* values in the rightmost column represent *p* group × time. * *p* < 0.05 vs. T1.

## Data Availability

Data are available from the corresponding author upon reasonable requests.
